# Evaluation of screening platforms for virus-like particle production with the baculovirus expression vector system in insect cells

**DOI:** 10.1038/s41598-020-57761-w

**Published:** 2020-01-23

**Authors:** Florian Strobl, Sahar Masoumeh Ghorbanpour, Dieter Palmberger, Gerald Striedner

**Affiliations:** 1 acib GmbH, Petersgasse 14, 8010 Graz, Austria; 2 Department of Biotechnology, University of Natural Resources and Life Sciences, Vienna, Austria

**Keywords:** Biomaterials - vaccines, Expression systems

## Abstract

Recombinant protein and virus-like particle (VLP) production based on the baculovirus expression vector system is fast, flexible, and offers high yields. Independent from the product, a multitude of parameters are screened during process development/optimisation. Early development acceleration is a key requirement for economic efficiency, and µ-scale bioreactor systems represent an attractive solution for high-throughput (HTP) experimentation. However, limited practical knowledge is available on the relevance and transferability of screening data to pilot scales and manufacturing. The main goal of the present study was to evaluate a HTP µ-bioreactor platform with respect to its aptitude as a screening platform mainly based on transferability of results to benchtop bioreactors representing the conventional production regime. Second question was to investigate to what extent the online sensors of the µ-bioreactor contribute to process understanding and development. We demonstrated that transferability of infection screening results from the HTP µ-bioreactor scale to the benchtop bioreactor was equal or better than that from shaker cultivation. However, both experimental setups turned out to be sub-optimal solutions that only allowed for a first and rough ranking with low relevance in the case of absolute numbers. Bioreactor yields were up to one order of magnitude higher than the results of screening experiments.

## Introduction

Over the last few decades, the baculovirus expression vector system (BEVS) has become a powerful tool for the production of a variety of recombinant proteins. More than 400 cell lines have since been modified to produce wild-type or recombinant baculovirus, recombinant proteins, virus-like particles (VLPs) or gene therapy vectors^1^. Parameters that have to be considered when infecting cells are the cell concentration at time of infection (CCI), the number of plaque-forming units per cell (PFU/cell) commonly described as multiplicity of infection (MOI), and the harvest time point. There are studies focused on development of mathematic models to calculate the best infection strategies including MOI, TOI, CCI and media depletion^2–4^. In these studies, *Sf9* cells expressing the same soluble secreted product were used. Over time insect cell lines from different species were established exhibiting growth, infection and production characteristics different from that of Sf9 cells. In several studies significant variation with respect to optimal MOI were observed, mostly stating that there are correlations between MOI levels and product formation or concentration. Recommended MOIs range from 1 to 20 plaque-forming units per cell^5^.

The CCI is another important factor influencing the infection efficiency, and CCIs >2 × 10^6^ cells mL^−1^ for *Sf9* and High Five lead to significantly reduced specific productivities, and even non-infected cell populations have been observed^6,7^. Another point to consider is the stability of the virus stock during long-term storage at 4 °C, which can lead to a decrease in virus titre^8^. In general, the determination of virus titre is a critical and time-consuming step and, independent of the methods used, there is significant analytical error in the range of ± 1 log fold changes^9^. This is valid for both plaque assay and tissue culture infectious dose 50 (TCID50), two methods commonly used and accepted in academia and industry^10^.

To identify the optimal CCI and MOI for high yield production of VLPs or proteins of interest, multiple costly and time-consuming cultivations have to be performed in small scale before transferring the process to a larger scale. In commonly used shake flasks or cell culture flasks, monitoring and control of key process parameters such as OD, pH and dissolved oxygen (DO) is limited or simply not possible. An alternative to conventional shaker flasks or cell culture flasks is the Biolector® (m2p-labs GmbH, Baesweiler, Germany), a titre plate-based platform. This high-throughout (HTP) µ-bioreactor system enables online monitoring of cell density, fluorescence, DO level, and pH in a continuously shaken 2.5 mL volume and has already been described as being well-suited for *Sf*9 insect cells^11^. Continuous scattered light measurement offers the possibility of obtaining real-time information on cell growth during cultivation. Moreover, fluorescence measurements can provide direct or indirect information on product formation and infection kinetics when the baculovirus harbours a fluorescence marker or the product is able to emit fluorescence itself. Biolector® was already used as a tool for condition screening to identify the optimal MOI and CCI for *Sf*9 insect cells producing recombinant secreted alkaline phosphatase. Identical behaviour was observed in TubeSpin® Bioreactor 50 experiments and the results transferred to stirred tank and wave bioreactors^12^. Nevertheless, there are systems used for other organism which, combined with shaker platforms, offer non-invasive measurement of OD^13^ and also pH and DO using shaker flasks^14^. For these systems special shaker flasks are needed and the incubator has to be adapted with a special platform which is connected to a control unit, with a limited number of shaker flasks per control unit (e.g. SFR vario (Presens).

However, for the BEVS, direct comparisons for transferability of results (cell growth, infection, and production) generated in screening experiments with the µ-bioreactor or shake flask to stirred tank bioreactor cultivations are missing. In this work, we summarise a benchmark study focused on the suitability and performance of the µ-bioreactor platform as a HTP screening tool for the early development and optimisation of BEVS. We varied the MOI in the µ-bioreactor, shake flask, and 1.5 L benchtop bioreactor cultivation scales and compared the results with respect to information content and transferability. We clearly demonstrate that the µ-bioreactor system is an efficient screening tool for baculovirus insect cell technology.

## Results and Discussion

Process knowledge is a key factor in bioprocess scale-up. In early stages of development, real-time process data are limited and, for shakers and adherent culture, little to no online monitoring tools exist. To close this gap, the Biolector®, an HTP µ-bioreactor system, was benchmarked as an alternative to shaker flasks, as it is capable of monitoring different process-relevant parameters online. As discussed transferability of results from shake flask experiments to bioreactors is limited the main focus of this study was to investigate if the µ-bioreactor system can serve as an alternative screening platform and to what extent generated results are transferable to benchtop bioreactors. Consequently, we did not use standard shake flask experiments as benchmark for the µ-bioreactor online sensor evaluation.

### Evaluation of online µ-bioreactor monitoring capabilities

In order to fully benefit from the online monitoring capabilities of the µ-bioreactor system, the output of each individual sensor during insect cell cultivation needs to be evaluated. The optodes for pH and DO measurement provide meaningful real-time information on the process state that can be used directly to describe and compare individual cultivations and to identify problems, such as DO limitations or critical pH values.

### Calibration of the light scatter signal

**Figure 1:**
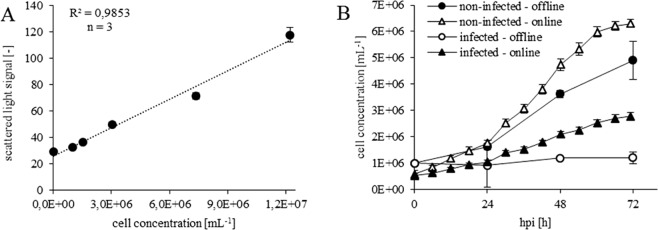
(**A**) Cell concentration calibration of the µ-bioreactor for Tnms42 cells. (**B**) Comparison of cell concentrations measured online and offline for non-infected and infected Tnms42 populations. The displayed mean values correspond to three different plate well titres (n = 3 and the error bars represent standard deviations). Online data recorded at a frequency of 15 minutes are displayed in 6 h interval to improve readability.

In general, the light scatter signal correlates with the cell concentration in the culture. However, calibration experiments are essential to identify this correlation for a respective cell system and cultivation regimen. To establish the calibration curve centrifuged cells were resuspended in media and diluted to respective concentrations. With the recommended shaking speed of 700 rpm for high insect cell concentrations^11^, we observed a high tendency of cells to settle and build up a layer on the bottom of the well. This led to a saturation of the scattered light signal, even at low cell densities, and consequently false cell count estimates. A previous study demonstrated that the scattered light signal directly correlates with a cell density of up to 3 × 10^6^ cells mL^−1^ using this shaking speed^15^. As the µ-bioreactor was established^16^ as a screening tool for bacterial cultivation with cell densities up to 10 g L^−1^, cell densities in standard BEVS cultivations should not lead to saturation of the light scattering signal. By increasing the shaking speed to 800 rpm, cell settling was eliminated and a direct correlation between the light scatter signals and cell concentrations up to 1.2 × 10^7^ cells mL^−1^ was demonstrated. However, the blank signal was quite high which can cause inaccurate estimates for low cell densities as the limit of detection is at least in the range of 1.0 × 10^6^ cells mL^−1^ (Fig. 1A). In Fig. 1B, the cell count calibration was tested on datasets from µ-bioreactor experiments. For both, infected and non-infected cells the calculated cell concentrations based on the calibration curve differed significantly from offline measured cell concentrations. The on-line values were lower in the early phase and higher in the later stage of the process. Several factors could contribute to the observed differences. The cells used for the calibration were concentrated via centrifugation and diluted to the corresponding concentrations with fresh media. In contrast to that treatment samples from cultivation experiments were directly measured in suspension. The supernatant changes continuously along the process due to cell lysis, vesicle formation, release of product and consumption of media compounds. Consequently, the background signal from samples can significantly differ from the calibration background signal and may lead to an overestimation of the cell concentration. Another source of variation is caused by changes in cell size during cultivation which can which can add up to more than 20% even in non-infected cultivations^17^. Finally, cell viability which is decreased to 57% for infected cultures and to 93% for non-infected cultures can also significantly influence the light scatter signal as dead cells show different light scattering properties.

### Evaluation of fluorescence for process monitoring

**Figure 2:**
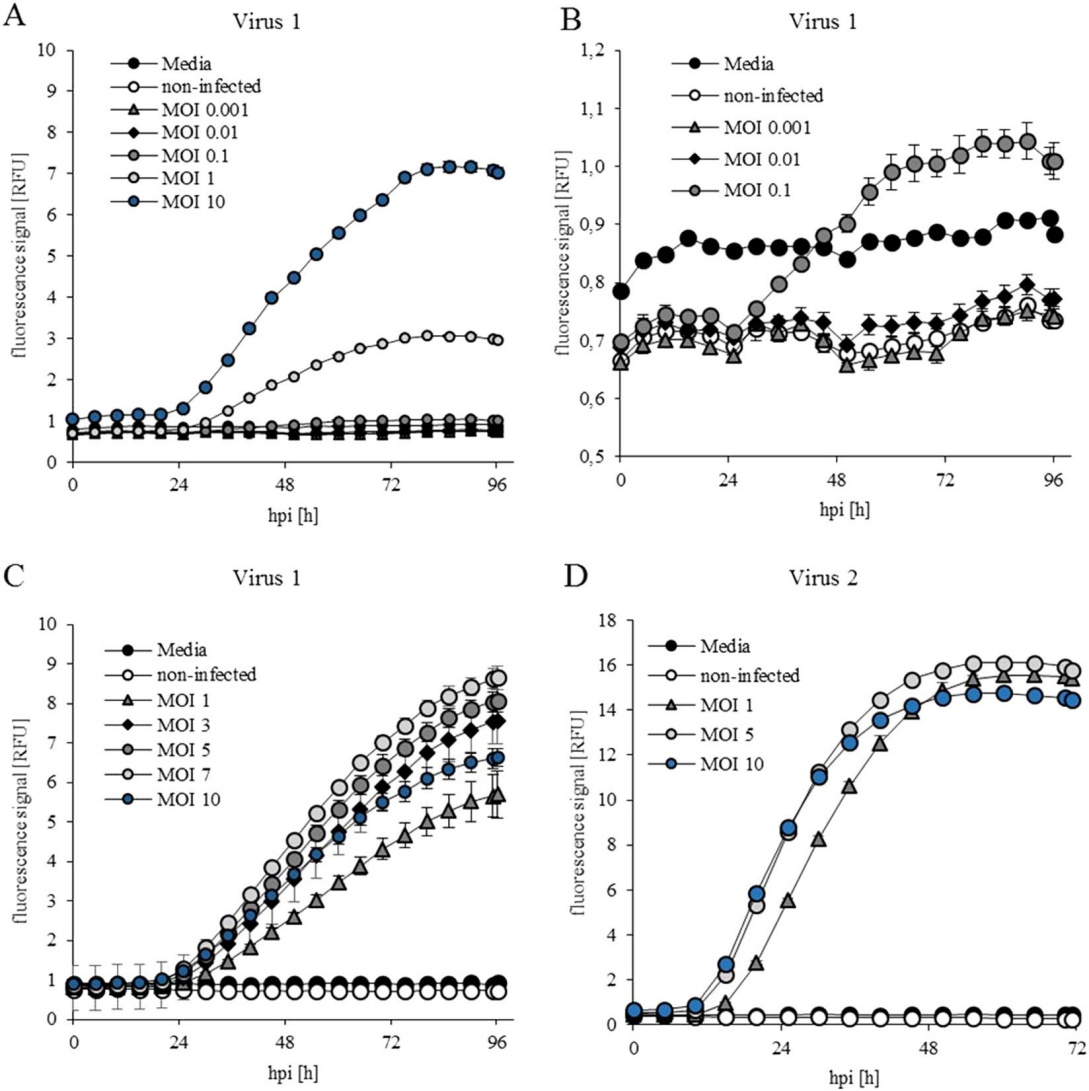
Fluorescence signals in non-infected and infected Tnms42 cultivations over time. (**A**) Experiment with Virus 1 with MOIs of 0.001 to 10 and (**B**) focused on MOIs ≤0.1 resulting in low fluorescence values. (**C**) Experiments with MOIs from 1 to 10. (**D**) Experiments with infection using Virus 2 using MOIs of 1 to 10. In each experiment, non-infected cells and media without cells were measured as references. All experiments were conducted in triplicate and the error bars represent standard deviations.

To evaluate the potential of the fluorescence measurements as a process-monitoring tool, a set of cultivations with different MOIs (0.001, 0.01, 0.1, 1, 3, 5, 7, and 10) were performed in the µ-bioreactor (Fig. 2A,C). For clarity of presentation, online data are displayed in 5-h intervals, though the measurement frequency was 15 min. The target product, H1Gag VLPs, was expressed using the same polyhedrin promoter as for yellow fluorescent protein (YFP), which was used as a marker of infection (Virus1). The process information delivered by the fluorescence signal measured online allowed for a first estimate of accurate MOI ranges and provided meaningful information on the starting point for protein production and infection efficiency. In experiments with MOIs <0.1, no YFP expression was observed (Fig. 2B) and an MOI of 0.1 resulted in an increase in the YFP signal after 24 hours. Within the MOI range of 0.1 to 7, the infection increase directly correlated with increased YFP signal and product formation. However, an MOI of 10 resulted in a reduced YFP signal (Fig. 2C).

The fluorescence curves provided additional information on infection and production kinetics. Production starts with a delay of approximately 18 hpi, independent of the applied MOI, and slopes are slightly steeper for higher MOIs. The information delivered by online fluorescence measurements represents a significant advantage of the µ-bioreactor over the shaker, with which fluorescence measurements are limited to offline samples.

Experiments with baculovirus in which YFP is under the control of the p6.9 promoter (Virus 2) showed that product formation had already started 11 hpi (Fig. 2D). This can be related to the p6.9 promoter, which starts production at an earlier stage than the polyhedron promoter and is described as a weaker promotor system^18^.

### Growth characteristics in the µ-bioreactor system and shake flask cultivation

**Figure 3:**
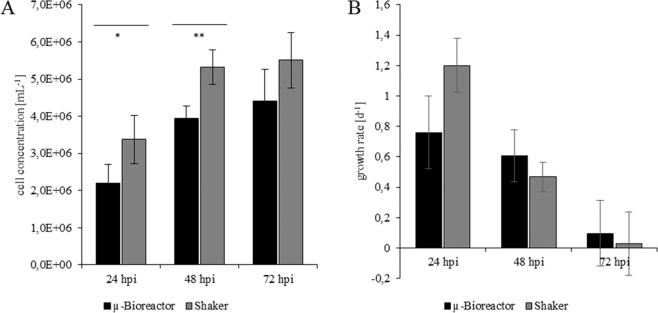
Comparison of Tnms42 cell cultivation in the µ-bioreactor (n = 5) and shake flasks (n = 4). (**A**) Cell densities and (**B**) growth rates per day displayed as mean with standard deviation. (**A**) Cell concentrations were analysed for normality by Shapiro-Wilk test and a t- test was performed, statistical significant differences are indicated (*P = 0.032, **P = 0.00258).

To evaluate the growth behaviour of cells in the µ-bioreactor system, we performed comparative experiments in shaker flasks using different passages of *Tnms42* cells. Cells were seeded at a cell density of 1.0 × 10^6^ cells mL^−^ and growth monitored via offline analysis (24 h sampling frequency) over 72 h without passaging or adding fresh media. The mean cell concentrations of four different shaker experiments and five different µ-bioreactor runs are given in Fig. 3A. The final *Tnms*42 cell concentrations in these experiments were comparable to literature values for *Tnms*42-related High Five cells grown under similar conditions^19,20^. The results in both cultivation regimens were comparable with regard to growth kinetics. However, cell growth in µ-bioreactor cultivations exhibited a delay that can be attributed to an initial phase of adaptation to µ-bioreactor conditions which was also seen when cells were transferred from shaker flasks into the benchtop bioreactor (see next section). There was a lower cell density and lower growth rate after 24 hours compared to shaker flask results (Fig. 3). At the end of cultivation, these significant differences were no longer present and similar growth kinetics observed with only 20% lower cell density in the µ-bioreactor system.

### Transferability of screening results to production environment and benchtop bioreactor conditions

The key question in process development is the extent to which results generated in screening experiments can be transferred to larger scales and production conditions. Based on growth and infection screening experiments in shaker flasks and µ-bioreactor cultivations, three different MOIs (MOI of 1, 5, and 10) were selected for a direct and more detailed comparison of the µ-bioreactor, shake flask, and benchtop bioreactor cultivations. As bioreactor experiments in 1.5 L and 15 L scale showed comparable growth kinetics and product yields (data not shown) we concluded that the 1.5 L scale is suited to generate results transferable to pilot scale of 50–100 L. *Tnms*42 cells were infected with the baculovirus for VLP production expressing YFP under control of the p6.9 promoter. Cultures were inoculated at a cell density of 1 × 10^6^ cells mL^−1^ with identical cell material from the same pre-culture. Shake flask and µ-bioreactor cultivations were infected at an MOI of 1, 5, and 10 using the same virus stock. The benchtop bioreactor cultivations were infected 24 hours after inoculation. In addition, non-infected control cultures were performed in each setting.

**Figure 4:**
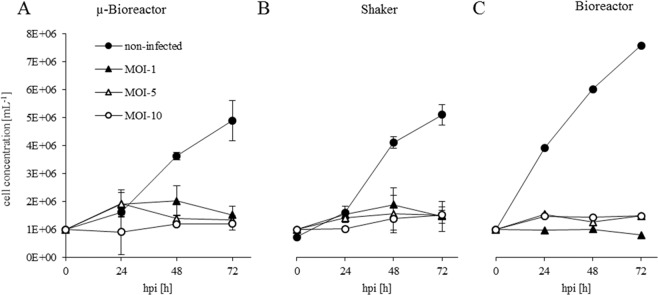
Course of the cell concentration of infected and non-infected Tnms42 cell cultures in (**A**) µ-bioreactor cultivation, (**B**) shake flask cultivation, and (**C**) benchtop bioreactor cultivation. All experiments in A and B were conducted in triplicate. Error bars indicate the standard deviation.

For non-infected cultures, the shaker and µ-bioreactor platforms achieved similar final cell concentrations of approximately 5 × 10^6^ cells mL^−1^ the DO level for the non-infected cultures never dropped below 70% (supplemented data), which was significantly lower compared to the final cell concentration of 7.5 × 10^6^ cells mL^−1^ generated in the stirred bioreactor with controlled DO and pH (Fig. 4). The differences in the final cell density of non-infected *Tnms*42 cultivations can be attributed mainly to an unfavourable pH in the shaker and µ-bioreactor cultures as described in the literature^21,22^. In benchtop bioreactor experiments, the pH was maintained at 6.4 ± 0.05. In contrast, the online pH value in the µ-bioreactor system increased from 6.45 to 6.65 in the first 20 hours, and then decreased during cultivation to 5.9 for the non-infected cells. Offline measurement of the shakers showed an increase in pH. At the starting point, the media in all shakers had a pH of 6.4, increasing to 6.8 after 24 hours and finally 7.0 at the end of the cultivation. The observed pH conditions in the screening set-ups were most likely inappropriate for insect cell cultivation. The pH values in the µ-bioreactor system cannot be used in combination with YFP-expressing cells, as the fluorescence signal interferes with the pH and OD measurement. Moreover, to evaluate the impact of pH on the growth behaviour of the insect cells, a microfluidic µ-bioreactor offering pH control could be used^23^.

**Figure 5:**
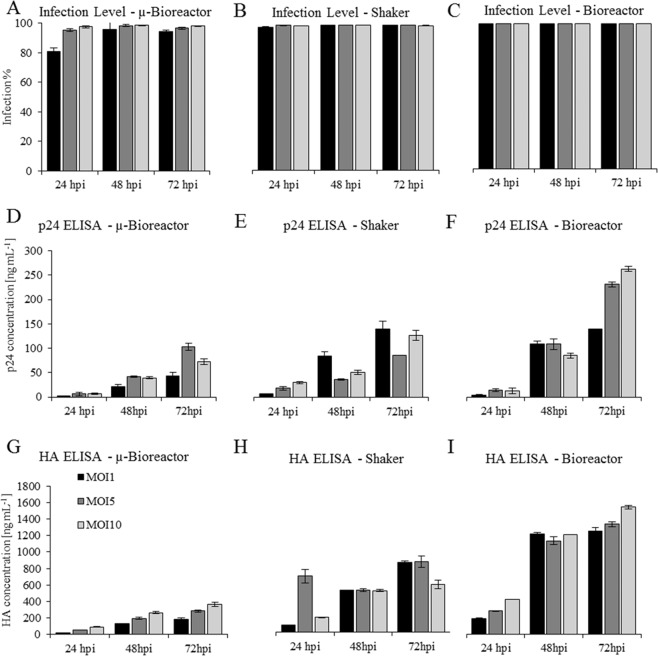
(**A**–**C**) The infection status of the three production systems over the course of the cultivations. (**D**–**F**) The hemagglutinin concentration. (**G**–**I**) The p24 concentrations measured with ELISA and standardised to 10^6^ cells mL^−1^ for all three cultivation platforms. The error bars indicate the standard deviation (n = 3).

For a more detailed characterisation of the infection status, offline samples were analysed using a flow cytometer, as each infected cell should produce YFP. The results clearly demonstrated that the infection efficiency was high in all settings with all MOIs (Fig. 5A–C). Independent from the MOI, all bioreactor and shaker cultivations were 100% infected after 24 hours. The µ-bioreactor performed similarly and reached the 100% infection level after 24 h in experiments with an MOI of 5 or 10. The major difference was observed for µ-bioreactor experiments with an MOI of 1, in which only 80% of the cells were infected after 24 hours. However, full infection was achieved after 48 hours in all cultivations with MOI = 1.

For identification of the optimal MOI and optimal length of production phase, HIV-p24-ELISA (Fig. 5D–F) and an influenza-HA-ELISA (Fig. 5G,H) were used to quantify VLP production over the course of the cultivations in the three platforms. Assuming a constant HIV-p24/HA ratio in produced VLPs, the two ELISAs resulted in identical rankings and characteristics with respect to product concentrations. This was the case for most of the samples except for the µ-bioreactor sample MOI 5/72 hpi and shaker flask sample MOI 5/24 hpi. As the variations in these samples cannot be attributed to analytical error, the two samples were not included in further interpretation of the results. With respect to the optimal length of the production phase, we observed increasing HIV-p24 and HA concentrations over time in all three platforms and throughout all infection levels (Fig. 5D–I). Consequently, 72 hpi has been shown to be the optimal production period for all cultivation setups under given infection and cultivation conditions. With respect to the MOI, the shake flask results yielded different rankings with an MOI of 1 as the preferred infection level.

Based on the assumption that a single VLP contains 5000 structural p24/GAG protein molecules^24,25^, a maximum of 8.8 × 10^8^ VLPs per 10^6^ insect cells was produced in the bioreactor with an MOI of 10. Experiments in both screening platforms yielded significantly lower specific VLP concentrations, with 5.27 × 10^8^ VLPs per 10^6^ cells in the shaker and 2.97 × 10^8^ VLPs per 10^6^ cells in the µ-bioreactor. Again, variations in pH in screening cultivations could be an important source of variation in product formation.

## Conclusion

In this work, the Biolector® µ-bioreactor system was evaluated as a platform for HTP insect cell culture cultivation and shown to be an attractive, cost-effective, and time-saving alternative to conventional shake flasks. With respect to provided online measurement capabilities the light scatter signal delivers information on cell growth but do not facilitate direct estimation of cell concentrations as there are inevitable error sources in real samples. The online fluorescence measurement delivers information on infection kinetics and efficiency if autofluorescent proteins like YFP are used as infection marker.The most important result was that the transferability of screening results from the µ-bioreactor to benchtop bioreactor, and that production conditions were acceptable with identical rankings and comparable to shaker flask cultivations. However, the use of both screening setups is limited regarding the estimation of final product titres because they are significantly lower than in benchtop bioreactor experiments. The experiments also revealed that *Trichoplusia ni* cell lines have a stronger influence on pH during cultivation than *Sf*9 cell lines, and that this variation may be a potential source of divergence in screening setups. In shake flasks, using medium with a stronger buffer system could be an option to improve the informative value of screening experiments, and the µ-bioreactor platform, a new system with microfluidics-based pH control, represents another possibility.

## Methods

### Insect cell lines

Spodoptera frugiperda 9 (*Sf*9) cells (ATCC CRL-1711) were used for the production of virus stock, and an alphanodavirus-free *Trichoplusia ni* - Tn5B1–4 (High Five) derivative, the *Tnms*42 cell line (BTI, Gary W. Blissard) for VLP production.

### Cloning and generation of recombinant baculoviruses and virus stock generation

Two different baculovirus working stocks were generated for the experiments. Virus1 encoded the nucleic acid sequence for the hemagglutinin (HA) 1 protein of Influenza A/California/04/2009 (H1N1) (GenBank accession no. JF915184.1), whereas Virus2 encoded the HA protein of Influenza A/Puerto Rico/08/1934 (H1N1) (GenBank accession no. EF467821.1). Both viruses encoded the matrix protein Gag of the type 1 human immunodeficiency virus (GenBank accession no. K03455.1). All genes were codon-optimised for expression in *Trichoplusia ni* and chemically synthesised by IDT (Leuven, Belgium). After PCR amplification, the HA of A/California/04/2009 was inserted into the pACEBac-1 acceptor vector (EMBL, Grenoble), resulting in pACEBac-1-H1; the HA of A/Puerto Rico/08/1934 was cloned into the pACEBac-2 acceptor vector, resulting in pACEBac-2-HA; and the Gag fragment was cloned into the pIDC donor vector (EMBL, Grenoble), resulting in pIDC-Gag. Cre-LoxP recombination of the acceptor and donor vectors resulted in H1Gag acceptor-donor fusion plasmids. The H1-Gag fusion plasmids were transformed into either *E. coli* DH10EMBacY (EMBL, Grenoble) or DH10EMBacp6.9Y, which harbour a YFP expression cassette under control of the polH or p6.9 promoter, respectively. Table 1 summarises the promoters used for gene expression in the two different viruses. The purified bacmid DNA was transfected into *Sf*9 cells using FuGene HD transfection reagent (Promega, Madison, Wisconsin, USA) according to the manufacturer’s instructions. The titre of the amplified passage 3 stock was determined by 50% tissue culture infective dose (TCID_50_).

**Table 1:** Promoters used for virus constructs.

	Virus1	Virus2
H1N1	polH	p10
Matrix Protein	polH	polH
Fluorescence Marker	polH	p6.9

### Cultivation strategies

#### Preculture

For all experiments, the *Tnms*42 cells were kept in exponential growth phase at 27 °C in shaker flasks at 100 rpm. The cells were grown in serum-free medium (Hyclone SFM4Insect, GE Healthcare) supplemented with 0.1% Kolliphor P188 (Sigma-Aldrich). Viable cell counts were determined by trypan blue exclusion using an automated cell counter (TC20 Biorad). For each experiment, cells were taken from adherent culture, transferred to suspension with a starting cell density of 0.5 × 10^6^ cells mL^−1^, and grown to desired cell numbers. All pre-cultures with *Tnms*42 cells were supplemented with heparin (1:1000) to avoid cell clumping.

#### µ-Bioreactor cultivation

The m2p Labs Biolector® is a micro-cultivation system enabling continuous online monitoring of cell density, fluorescence, DO levels, and pH in a continuously shaken microtitre plate format. Up to 48 different cultures were performed in parallel in one experiment in deep well, round plates equipped with optodes for DO and pH measurement. The working volume was 1.7 mL, the shaking speed 800 rpm, the temperature 27 °C, and the humidity 85%. Plates were inoculated and infected simultaneously with an initial cell concentration of 1 × 10^6^ cells mL^−1^ without any further passaging during the experiment. All experiments were performed in triplicate.

#### Shaker flask cultivation

Shaker flask cultivations were performed to generate an adequate reference data set following the conventional standard procedure. The cells were cultivated in triplicate in 200 mL shake flasks at a working volume of 20 mL. The cultures were inoculated with an initial cell density of 1 × 10^6^ cells mL^−1^ and infection performed simultaneously with the inoculation. The temperature of the incubator was set at 27 °C and 100 rpm.

#### Benchtop bioreactor cultivations

Experiments were performed in a 1.5 L bioreactor (DASGIP SR1500 DLS, Eppendorf) equipped with three Rushton impellers. The temperature was set to 27 °C and the pH maintained at 6.4 ± 0.05 using 25% (v/v) phosphoric acid and 7.5% (w/v) sodium bicarbonate. The DO level was maintained at 30%. Cells were inoculated at a cell density of 1 × 10^6^ cells mL^−1^ and cultivated in the bioreactor for 1 day prior to infection. Cell counts in the four bioreactors were determined, and each vessel was infected with the respective amount of virus and simultaneously diluted back to 1 × 10^6^ cells mL^−1^.

### Infection strategy and sampling

Cells in all three cultivation platforms were infected at an MOI of 1, 5, and 10, and one culture of non-infected cells was grown in parallel. As the virus stock is stored at 4 °C and studies have shown that the titre decreases over time^8^, a sample was analysed to determine the TCID50 of the virus stock. Sampling was performed over a period of 72 hours at 24-hour intervals.

### Analytical methods

#### Flow cytometry

To evaluate the infection status on a single cell level, a CytoFlex flow cytometer (Beckman Coulter Life Sciences) was used to discriminate between infected and non-infected cells. A total of 1 mL of cell suspension was centrifuged for 5 minutes at 300 × g, the supernatant frozen at -20 °C for further testing, and the pellet washed once in 1x phosphate-buffered saline (PBS). They cytometer was equipped with a 488 nm laser, enabling excitation of YFP. We recorded 10000 events per sample and used Kaluza software (Beckman Coulter version 2.1) for the data analysis.

#### Tissue culture infectious dose 50 (TCID50) assay

The titre of virus stocks was determined using TCID50^26^ based on the detection of YFP fluorescence. *Sf*9 cells were infected with serial dilutions of virus stock or supernatant samples of the different cultivations in a 96-well culture plate (Corning Incorporated, USA) and incubated at 27 °C without agitation. After 4 days, the wells were inspected for fluorescence using a fluorescence microscope (Leica DMIL-LED).

#### Enzyme-linked immunosorbent assay (ELISA)

The HA content in the expression supernatant was determined using the Influenza A H1N1 (A/Puerto Rico/8/1934) Hemagglutinin/HA ELISA pair set (Sino Biological, Wayne, USA) according to the manufacturer’s recommendations. For solubilisation of VLP-incorporated HA surface glycoproteins, samples were pre-treated with 1% zwitterionic detergent 1% (w/v) (Zwittergent 3–14, Calbiochem, San Diego, CA) for 30 minutes at room temperature^27^. A soluble trimeric insect cell expressing HA protein served as the calibration standard^28^.

The HIV-Gag polyprotein was indirectly quantified by measuring the concentration of p24, the major viral core structural protein generated by viral protease cleavage of Gag. Free soluble HIV-1 p24 and total HIV-1 p24 concentration, including VLP-incorporated p24, in the expression supernatant were determined by the HIV-1 p24 capsid protein p24 ELISA Kit (Sino Biological, Wayne, USA). For measurement of the total p24 concentration, VLPs were disrupted by incubation with SNCR buffer for 10 min at 70 °C, followed by an incubation step in 0.5% (v/v) Triton X-100 for 10 min at 99 °C^29^.

The influenza HA and HIV-1 p24 ELISAs were both developed with 100 µL of SIGMAFAST™ OPD substrate (Sigma Aldrich, St. Louis, MO, USA), and the reaction was stopped by the addition of 50 µL 3 N H2SO4 solution. The absorbance was measured at 492 nm and 620 nm (reference wavelength) using a Tecan Infinite 200 Pro (Tecan, Männedorf, CH) and data fitted to a 4th degree polynomial equation of a duplicate calibration curve.

### Statistical analysis

SigmaPlot 13 software was used for statistical analysis. Shapiro-Wilk test was used for normality distribution and t-test was used for comparison of differences between groups. The calculated probability (p) values were two-tailed, differences were considered as statistically significant if the p-value was lower than 0.05.

## Supplementary Information

Supplementary data
