# Supplementary material for: Evaluation of screening platforms for virus-like particle production with the baculovirus expression vector system in insect cells
Source: Sci Rep. 2020 Jan 23;10:1065. doi: 10.1038/s41598-020-57761-w (PMC6978312; doi:10.1038/s41598-020-57761-w)
Supplement: Supplementary file 1 — Supplementary data [file 41598_2020_57761_MOESM1_ESM.pdf]

Evaluation of screening platforms for virus-like particle production with the baculovirus expression vector system in insect cells

Florian Strobl [ab], *Sahar Masoumeh Ghorbanpour* [ab] *Dieter Palmberger* [ab] *and Gerald Striedner*[b*]

[a] *acib GmbH, Petersgasse 14, 8010 Graz*

[b] *Dep. of Biotechnology, University of Natural Resources and Life Sciences, Vienna, Austria*

* Correspondence to [gerald.striedner@boku.ac.at]

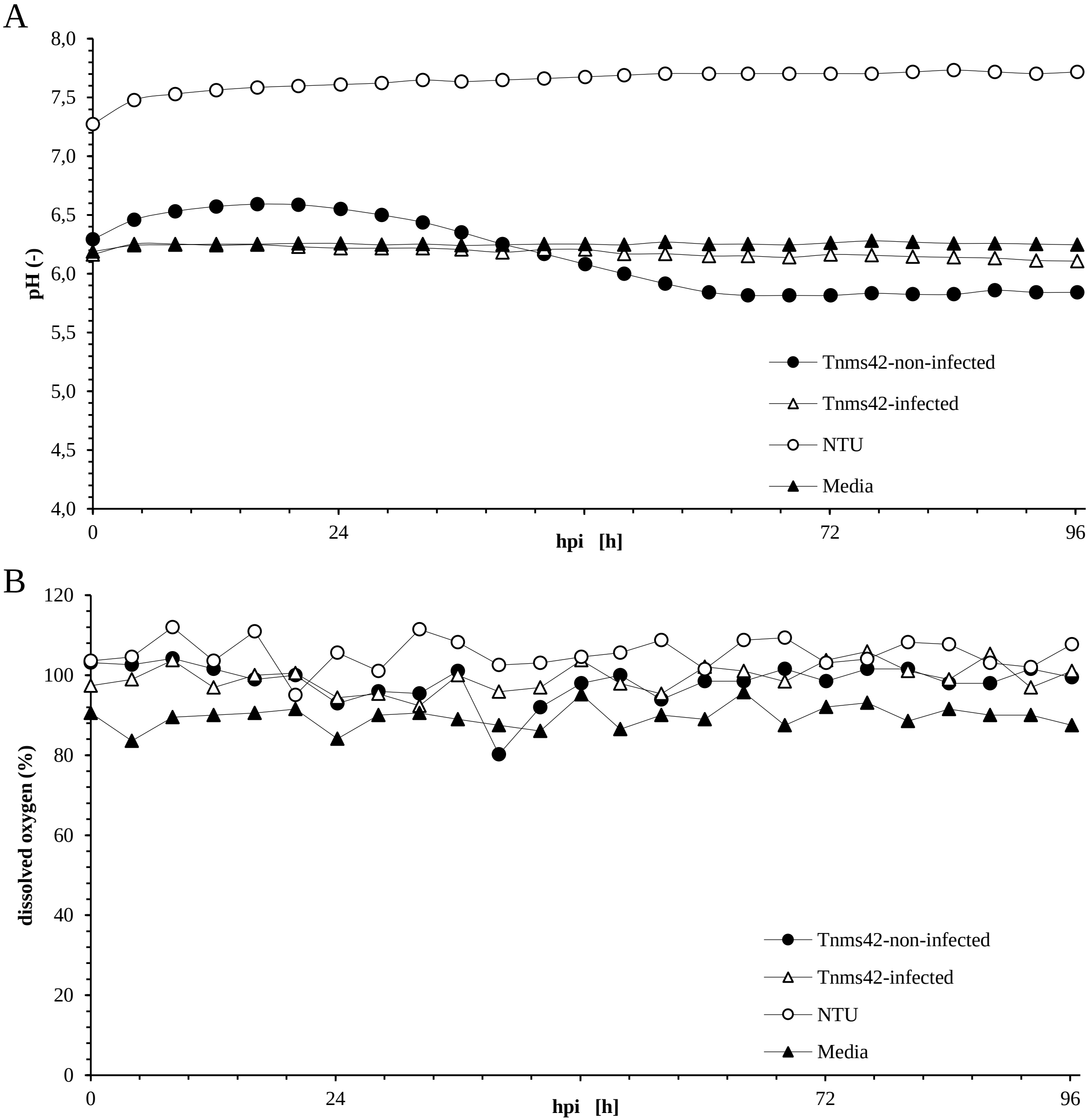
A
8,0
7,5
7,0
6,5
6,0
5,5
5,0
4,5
4,0
pH (-)
0
24
72
96
hpi [h]
Tnms42-non-infected
Tnms42-infected
NTU
Media
B
120
100
80
60
40
20
0
dissolved oxygen (%)
0
24
72
96
hpi [h]
Tnms42-non-infected
Tnms42-infected
NTU
Media

Figure 6: pH (**A**) and DO (**B**) time courses of infected non-infected Tnms42 cells, media and NTU200 standard are displayed. A virus without a fluorescence marker was used to demonstrate the pH and DO behavior over time.
